# Density and structure of DNA immobilised on gold nanoparticles affect sensitivity in nucleic acid detection

**DOI:** 10.1038/s41598-025-92474-y

**Published:** 2025-03-10

**Authors:** Nanami Fukuzumi, Gen Hirao, Atsushi Ogawa, Tsuyoshi Asahi, Mizuo Maeda, Tamotsu Zako

**Affiliations:** 1 Department of Chemistry and Biology, Graduate School of Science and Engineering, Ehime University, 2-5 Bunkyo, Matsuyama, Ehime 790-8577 Japan; 2 Proteo-Science Center, Ehime University, 2-5 Bunkyo, Matsuyama, Ehime 790-8577 Japan; 3 RIKEN Cluster for Pioneering Research, 2-1 Hirosawa, Wako, Saitama 351-0198 Japan

**Keywords:** Gold nanoparticles, Aggregation, Nucleic acid detection, Dispersion stability against salt, Density and structure of immobilised DNA, Bioanalytical chemistry, Colloids

## Abstract

Gold nanoparticles (AuNPs) are used as colorimetric biosensors that, combined with immobilised single-stranded DNA (ssDNA-AuNPs), can be used in genetic diagnosis because of their rapid and sequence-specific aggregation properties. Herein, we investigated the effect of the steric structure and density of immobilised DNA on AuNPs in non-crosslinking aggregation-based nucleic acid detection. Detection sensitivity improved with decreasing DNA density for linear conformations, but worsened for those with more rigid stem structures. We controlled the density of immobilised DNA using two different methods and investigated the aggregation behaviour of ssDNA-AuNPs. Interestingly, controlling the immobilised DNA density through ethylene glycol treatment had different effects on ssDNA-AuNP aggregation compared to those of alkanethiol substitution. This study suggests that the sensitivity of ssDNA-AuNPs for detecting target DNA could be affected by density and structure of the immobilised DNA.

**Supplementary Information:**

The online version contains supplementary material available at 10.1038/s41598-025-92474-y.

## Introduction

Gold nanoparticles (AuNPs) have been utilised as sensors because of their unique chemical and physical properties, which vary with size and shape^1–4^. AuNP solutions change colour from red to blue upon aggregation due to a change in surface plasmon coupling. The surface of AuNPs can be modified to aggregate upon binding to/detecting a target molecule. Various AuNP-based colorimetric sensors have been developed to detect DNA, proteins, metal ions, and organic/inorganic molecules^2,5–11^. In previous studies, duplex formation between immobilised single-stranded (ss)DNA (probe DNA) on AuNPs and target complementary ssDNA (target DNA) resulted in salt-induced aggregation mainly due to stacking forces between the blunt ends of the formed double strands^12,13^. This inspired the development of nucleic acid detection methods based on non-crosslinking aggregation using ssDNA-immobilised AuNPs (ssDNA-AuNPs), which has been applied in genetic diagnosis due to its rapid and sequence-specific aggregation properties^14–16^.

**Fig. 1:**
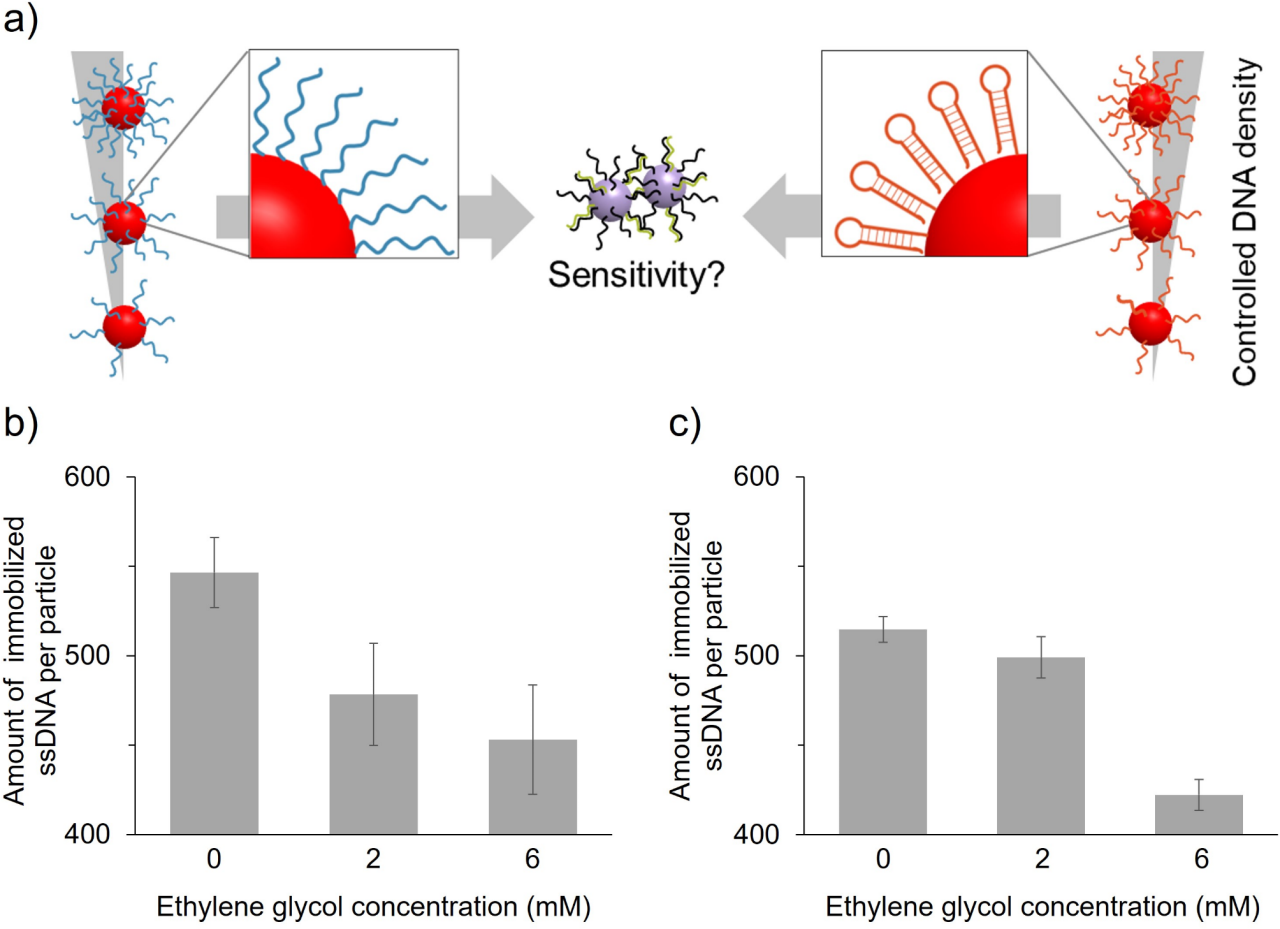
(**a**) Effect of probe DNA density on detection sensitivity using non-crosslinking aggregation of ssDNA-AuNPs. Two structural types of immobilised probe DNA were used. (b and c) Effect of EG on the densities of probe DNA-1 (**b**) and DNA-2 (**c**) immobilised on AuNPs. Averaged values of the three independent measurements are shown.

The effects of the thickness and steric structure of probe DNA layers on the dispersion stability and detection sensitivity of ssDNA-AuNPs have been investigated^17–20^. Our group developed a method to control the density of probe DNA immobilised on the surface of AuNPs using ethylene glycol (EG), which is expected to control the ice crystal spacing during freeze-thaw ssDNA-AuNP synthesis (Freeze method)^10^. We also investigated the effect of probe DNA density on the sensitivity of target DNA detection and showed that lower densities were associated with improved detection sensitivity. However, the structure of surface-immobilised DNA affects the aggregation of ssDNA-AuNPs with reduced colloidal stability against salt^18^. Therefore, this study investigated the effect of DNA density on the dispersion stability of ssDNA-AuNPs retaining a stem-loop structure and sensitivity of target DNA detection (Fig. 1a). We found that detection sensitivity was improved under reduced densities of linear probe DNA, but worsened when using probe DNA with a rigid stem structure. So far, ssDNA-AuNPs using structure-preserving ssDNA have been applied to molecular detection in various ways. For example, sensitive DNA detection method combining FRET and ssDNA has been reported, in which ssDNA retaining a stem-loop structure is used^21^. In addition, colorimetric sensor using AuNPs modified with DNA aptamer retaining the G-quadruplex structure^22^ and one using a non-crosslinking aggregation of ssDNA-AuNPs following the conformational change of immobilized aptamer^23^ have been reported. However, effect of the structure of the probe DNA on the detection sensitivity of complementary target DNA has not been investigated. Thus, this study will be a basis for further development of nucleic acid detection by ssDNA-AuNP using various sequences.

We also investigated the effect of alkanethiol treatment on the aggregation of ssDNA-AuNPs. Alkanethiols have thiol groups that promote monolayer formation and immobilised DNA displacement on Au surfaces and can thus be used to control the probe DNA density^24,25^. Our result indicated that ssDNA-AuNPs treated with alkanethiol showed different aggregation behaviour from those treated with EG. This was likely due to physical adsorption of the DNA backbone on the AuNP surface and upright structure of the immobilised DNA caused by the alkanethiol surface modification. Therefore, the sensitivity of ssDNA-AuNPs for detecting target ssDNA may be affected by the immobilised DNA density and choice of density control method. This study provides an important framework for determining the optimum DNA immobilisation conditions for the development of ssDNA-AuNP sensors.

## Results and discussion

### Control of immobilised density of two types of probe DNAs

We investigated two types of probe and target DNAs (Table 1). Probe DNA-1 is an ssDNA with a complementary sequence to target DNA-1 [a model biomarker that has an identical DNA sequence with micro RNA 145 (miR145), a biomarker for various cancers^26^], whereas probe DNA-2, with a similar ssDNA length compared to DNA-1, includes an 8-bp rigid stem-loop structure (ESI, Fig. S1). These two probe DNAs were immobilised on the surface of the AuNPs (type-1 and type-2, respectively) via thiol-Au bonds using the freeze method, and the immobilised probe DNA densities were controlled using EG, as previously reported^10,27,28^. Figure 1b and c show the densities of immobilised probe DNA-1 and DNA-2, respectively. For both sequences, the amount of immobilised DNA decreased in an EG concentration-dependent manner, consistent with our previous finding using other probe DNA with different sequences^10^, indicating the applicability of this density control method for DNA with a stem conformation.

**Table 1:** Sequences of ssDNAs

DNA	Sequence
probe DNA-1	5′-SH-(CH_2_)_6_-AAG GGA TTC CTG GGA AAA CTG GAC-3′
probe DNA-2	5′-SH-(CH_2_)_6_-AT**A GAG GTT G**GA GCT **CAA CCT CT**C-3′
target DNA-1	5′-GTC CAG TTT TCC CAG GAA TCC CTT-3′
target DNA-2	5′-GAG AGG TTG AGC TCC AAC CTC TAT-3′

The sequences considered to form the stem structure are bold.

### Effect of DNA density on dispersion stability of ssDNA-AuNPs against salt

**Fig. 2:**
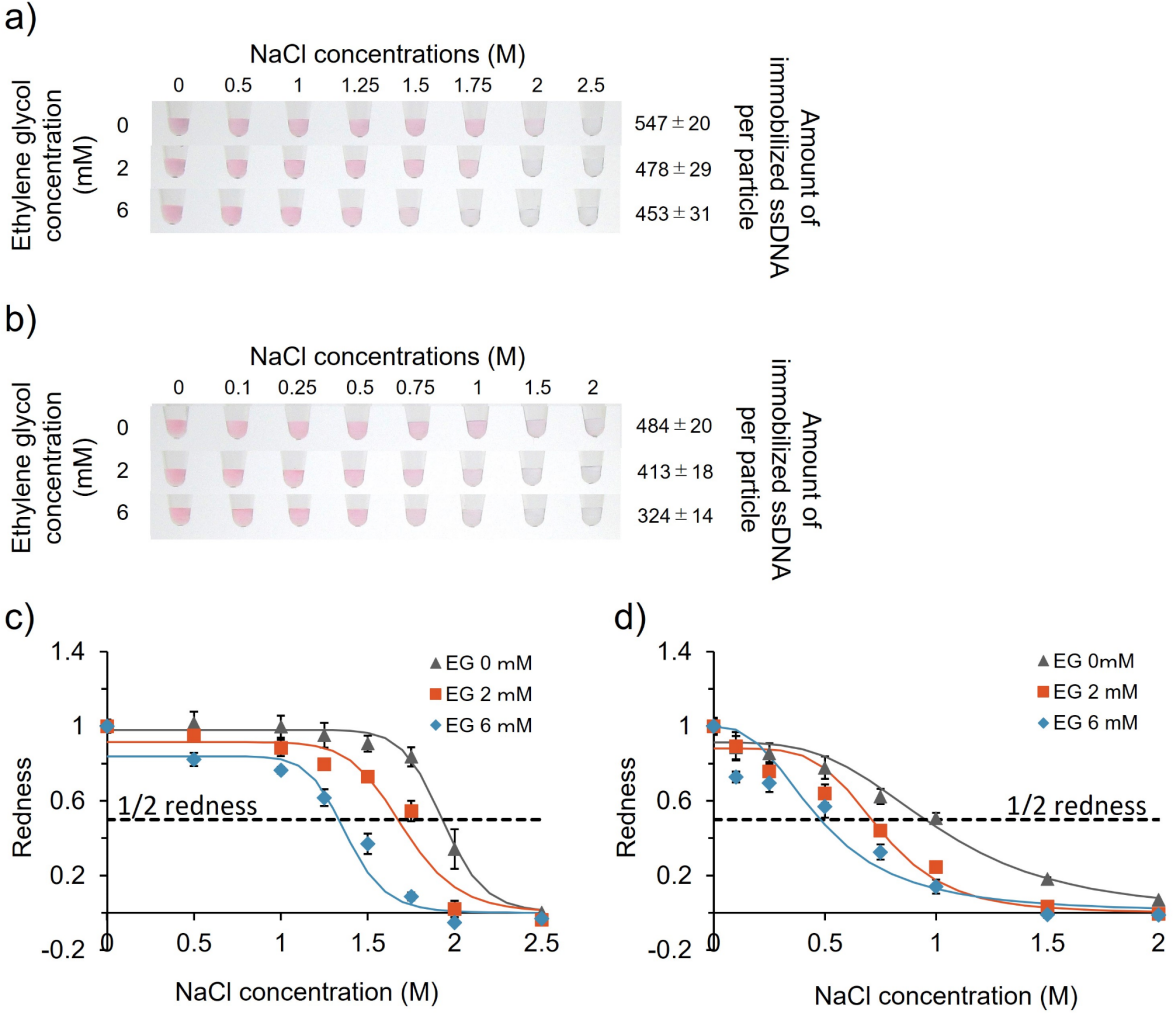
Effect of immobilised probe DNA density on the dispersion stability of ssDNA-AuNPs against salt. (**a** and** b**) Colour of AuNP solutions [type-1 (a), type-2 (b)] under increasing concentrations of NaCl. ssDNA-AuNP samples synthesised in the presence of different concentrations of EG (left) are shown. The number of immobilised ssDNA per particle is shown on the right. (**c** and** d**) Normalised redness values for type-1 (**c**) and type-2 (**d**) samples. The redness values of samples without NaCl were normalised to 1.0. Averaged values from three different samples are shown. C_½ redness_: (**c**) 1.93 M (EG 0 mM), 1.67 M (EG 2 mM), 1.33 M (EG 6 mM), (**d**) 0.951 M (EG 0 mM), 0.710 M (EG 2 mM), 0.478 M (EG 6 mM).

We evaluated the effect of immobilised probe DNA density on the dispersion stability of ssDNA-AuNPs against salt. Specifically, we analysed the colour of the ssDNA-AuNP solution upon salt addition, which induces aggregation by reducing electrostatic repulsion. The colour of the solution changed from red to blue/clear with increasing NaCl concentration (Fig. 2a and b). The NaCl concentration required to change the colour of the solution was lower at lower DNA densities, consistent with our previous finding using other DNA^10^, indicating that the dispersion stability of ssDNA-AuNPs in the presence of NaCl worsened with decreasing immobilised DNA density. Colour changes were also quantitatively estimated using a redness parameter obtained from the intensity of each colour component as previously described^10^. Figure 2c and d show the redness values for type-1 and type-2 samples, respectively, supporting the DNA density-dependent aggregation behaviour of ssDNA-AuNPs against NaCl. Differences in the dispersion stability among ssDNA-AuNPs with different probe DNA densities were also supported by the statistical analysis (ESI, Fig. S2).

### Effect of DNA density on target ssDNA detection using ssDNA-AuNPs

**Fig. 3:**
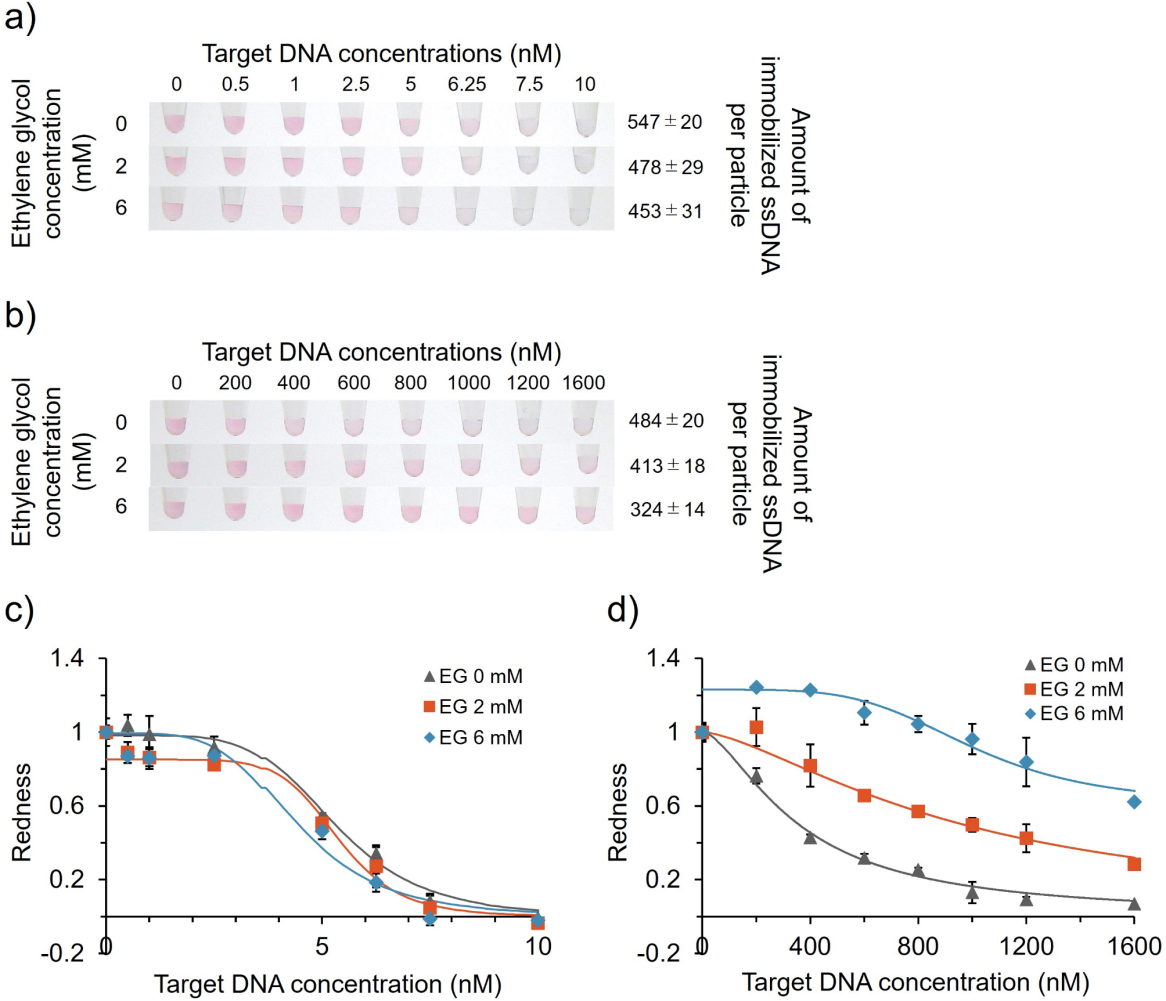
Effect of immobilised probe DNA density on the sensitivity of ssDNA-AuNPs for target DNA detection. (a and b) Colour of AuNP solutions upon addition of increasing concentrations of target DNA-1 (**a**) and DNA-2 (**b**). The ssDNA-AuNP samples synthesised in the presence of different concentrations of EG (left) are shown. The number of immobilised ssDNA per particle is shown on the right. (c and d) Normalised redness values for type-1 (**c**) and type-2 (**d**) samples. The redness values of samples without target DNA were normalised to 1.0. Averaged values from three different samples are shown. LOD: (**c**) 3.36 nM (EG 0 mM), 3.84 nM (EG 2 mM), 1.76 nM (EG 6 mM), (**d**) 103 nM (EG 0 mM), 183 nM (EG 2 mM), 1120 nM (EG 6 mM).

We investigated the effect of immobilised DNA density on the detection sensitivity of target DNA based on the changes in colour/ssDNA-AuNP aggregation induced by duplex formation of the target and probe DNAs in the presence of salt. The colour of the solution changed from red to blue/clear as the amount of target DNA increased (Fig. 3a), indicating successful detection. AuNPs with a lower DNA density were more sensitive (Fig. 3a), consistent with previous results using other DNA^10^. However, for the type-2 sample, AuNPs with a higher DNA density were more sensitive (Fig. 3b). These observations were supported by the redness parameters (Fig. 3c and d). Differences in the detection sensitivity among ssDNA-AuNPs with different probe DNA densities were also supported by the statistical analysis (ESI, Fig. S2). Limit of detection (LOD) values were also calculated using 3σ criterion method as the concentration that gives lower value than 3σ line (= background + 3σ), where σ denotes the standard deviation of zero-concentration background data (ESI, Fig. S3) as previously reported^29^. The obtained LOD values were 3.36 nM (EG 0 mM), 3.84 nM (EG 2 mM), 1.76 nM (EG 6 mM) for type-1 and 103 nM (EG 0 mM), 183 nM (EG 2 mM), 1120 nM (EG 6 mM) for type-2, respectively. These results indicated that the type-1 and type-2 ssDNA-AuNPs have different behaviours in terms of structure of immobilised probe DNA, which affects their sensitivity.

### Effect of DNA density on size and duplex formation ratio of ssDNA-AuNPs

**Fig. 4:**
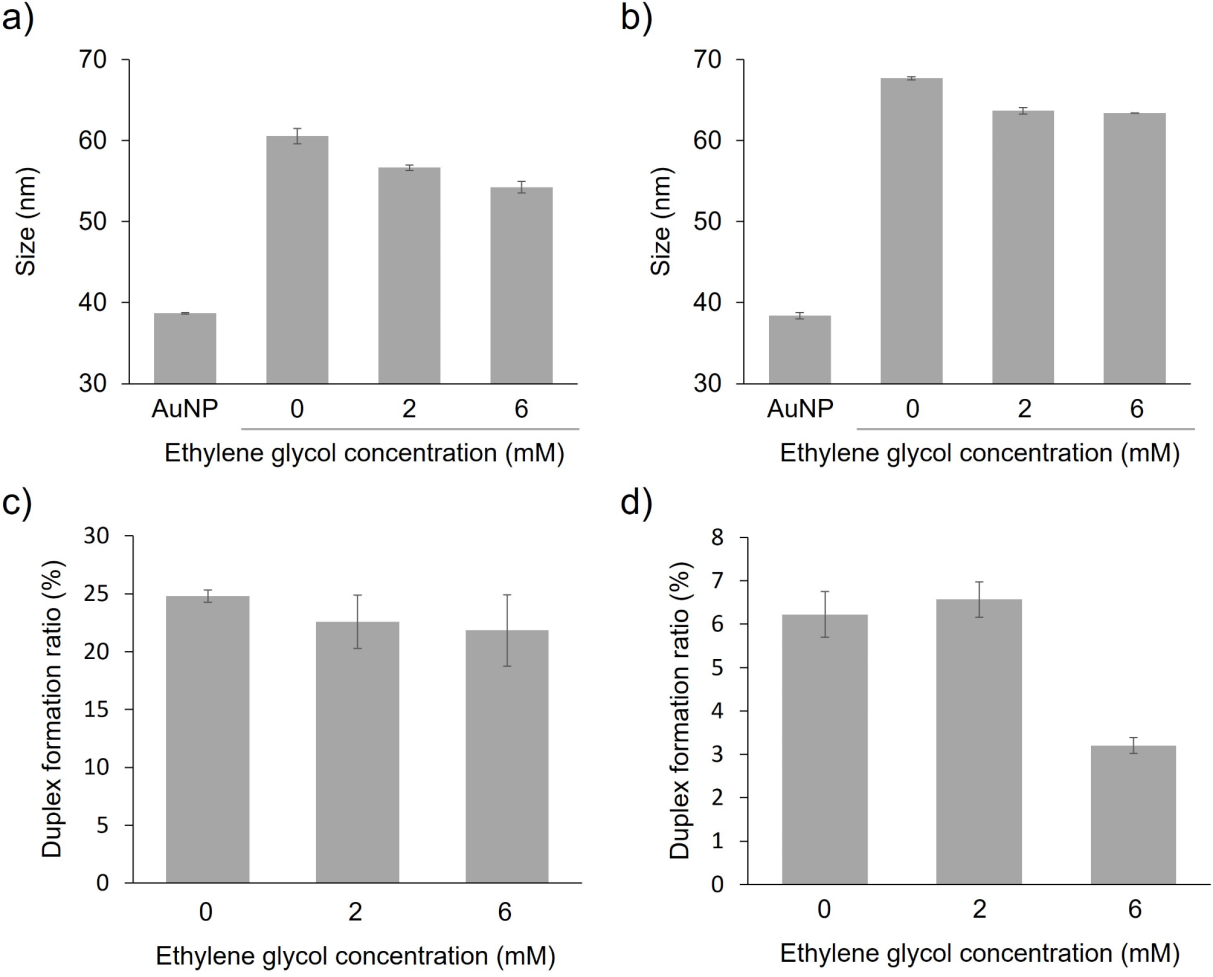
(**a** and** b**) Effect of density on type-1 (**a**) and type-2 (**b**) particle sizes in the presence of EG. (**c** and** d**) Effect of density on type-1 (**c**) and type-2 (**d**) duplex formation. Averaged values from three measurements are shown.

We evaluated the mechanism associated with the variation in dispersion stability and detection sensitivity at different immobilised probe DNA densities based on the particle size and duplex formation ratio of the ssDNA-AuNPs. The particle sizes of both types of ssDNA-AuNPs decreased with decreasing amounts of immobilised ssDNA (Fig. 4a and b). The thickness of immobilised ssDNA on nanoparticle surfaces is expected to decrease with decreasing ssDNA densities^30,31^. Low-density immobilised ssDNA adopts a more randomly oriented structure on AuNPs, whereas those at high densities adopt a more upright structure^30,31^. Steric stabilisation, which is related to the thickness of the DNA layer, strongly affects the stability of ssDNA-AuNPs^19^. Therefore, we hypothesised that the decrease in DNA density reduced the electrostatic repulsive force and steric hindrance derived from the immobilised DNA, resulting in reduced dispersion stability for both type-1 and type-2. Figure 4c and d show the duplex formation ratios of the target and probe DNAs for type-1 and type-2, respectively. Interestingly, type-2 had a lower duplex formation ratio than type-1, especially under low-density conditions (i.e. high EG concentrations), and the duplex formation ratio decreased significantly to 3.2 ± 0.18%. Based on the changes in particle size (Fig. 4b) and the structural propensity of probe DNA-2, it was suggested that the stem-loop structure is formed for ssDNA-AuNPs with lower probe DNA density. Thus, it is plausible that duplex formation in type-2 was reduced for ssDNA-AuNPs with lower density due to the formation of the stem-loop structure. Similarly, AuNP-immobilised DNA aptamers exhibited better folding at lower densities^18^. For type-1, the dispersion stability of ssDNA-AuNPs reduced, while the duplex formation capacity was maintained, i.e. the loss of stability against salt and the attraction force between the blunt-ends of immobilised dsDNA^13^ may have improved detection sensitivity at lower ssDNA densities. In contrast, in type-2, attraction force between particles decreased at lower ssDNA densities owing to the reduced duplex formation, causing decreased detection sensitivity regardless of the decrease in dispersion stability.

### Effect of 6-mercapto-1-hexanol on probe DNA density and AuNP aggregation behaviour

**Table 2:** Sequences of probe and target DNA-3

DNA	Sequence
Probe DNA-3	5′-SH-(CH_2_)_6_-TCA CAG GTT AAA GGG TCT CAG GGA-3′
Target DNA-3	5′-TCC CTG AGA CCC TTT AAC CTG TGA-3′

**Fig. 5:**
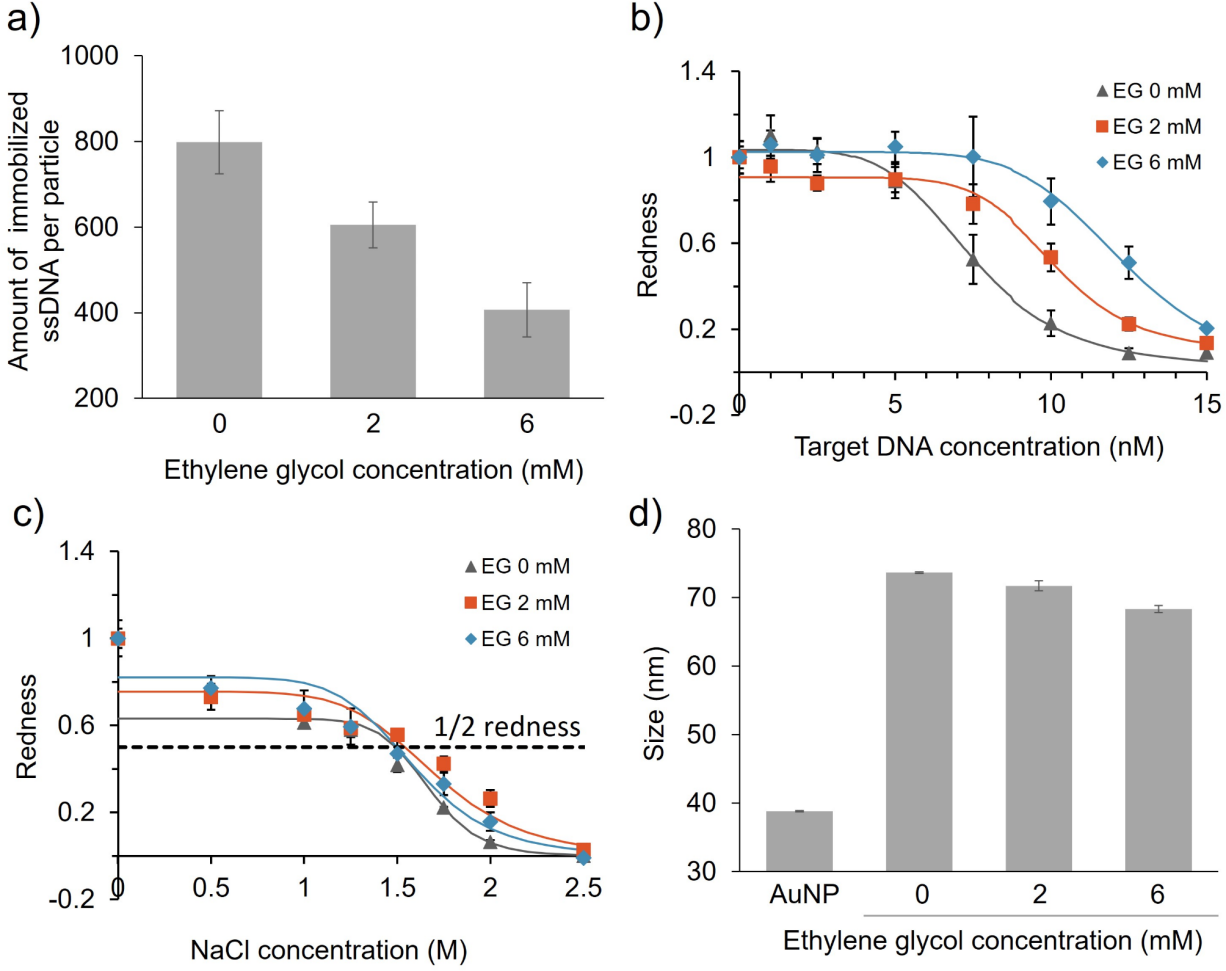
Control of immobilised probe DNA-3 density using EG and effect on type-3 ssDNA-AuNP aggregation. (**a**) Density of probe DNA-3 immobilised with EG. Averaged values from three independent measurements are shown. (**b** and **c**) Effect of immobilised probe DNA density on detection sensitivity (**b**) and dispersion stability of ssDNA-AuNPs against salt (**c**). Normalised redness values obtained from the solution colour. The redness value of samples without NaCl or target DNA was normalised to 1.0. Averaged values from three different samples are shown. LOD: 5.97 nM (EG 0 mM), 8.10 nM (EG 2 mM), 9.85 nM (EG 6 mM). C½ redness: 1.49 M (EG 0 mM), 1.54 M (EG 2 mM), 1.50 M (EG 6 mM). (**d**) Effect of density on ssDNA-AuNP particle size. Averaged values from three measurements are shown.

We investigated the effect of immobilised DNA density on the aggregation of AuNPs bound to probe DNA-3 (type-3), which has a sequence complementary to that of miR125a, a biomarker for oral cancer (target DNA-3, Table 2)^32^, which is thought to have a linear structure similar to DNA-1 and the DNA used in the previous study^10^. The amount of probe DNA-3 immobilised on AuNPs decreased in an EG concentration-dependent manner (Fig. 5a), consistent with the results for type-1 and type-2 (Fig. 1b and c). Interestingly, in contrast to type-1, the detection sensitivity for target DNA worsened with decreasing probe DNA density (Fig. 5b) (LOD=5.97 nM (EG 0 mM), 8.10 nM (EG 2 mM) and 9.85 nM (EG 6 mM)). Change in the dispersion stability against salt was not significant for the samples with different probe DNA density (Fig. 5c), unlike for type-1 and type-2. To elucidate the mechanism associated with these differences, we assessed the particle size and zeta potential of the type-3 ssDNA-AuNPs. Particle sizes decreased with a reduction in DNA density (Fig. 5d). Zeta potential also decreased at lower densities, but only for type-3 (ESI, Fig. S4). ssDNA is known to interact with and adsorb to the Au surface^33–36^. Moreover, it was reported that AuNPs modified with unfolded aptamers are less stable than AuNPs modified with folded aptamers because of a decrease in the thickness of the surface DNA layer in the presence of salt, given that unfolded aptamers shrink more than folded/structured aptamers^18^. Taken together, we hypothesised that low-density probe DNA-3 may also be partially adsorbed onto the Au surface and maintain the thickness of the DNA layer.

**Fig. 6:**
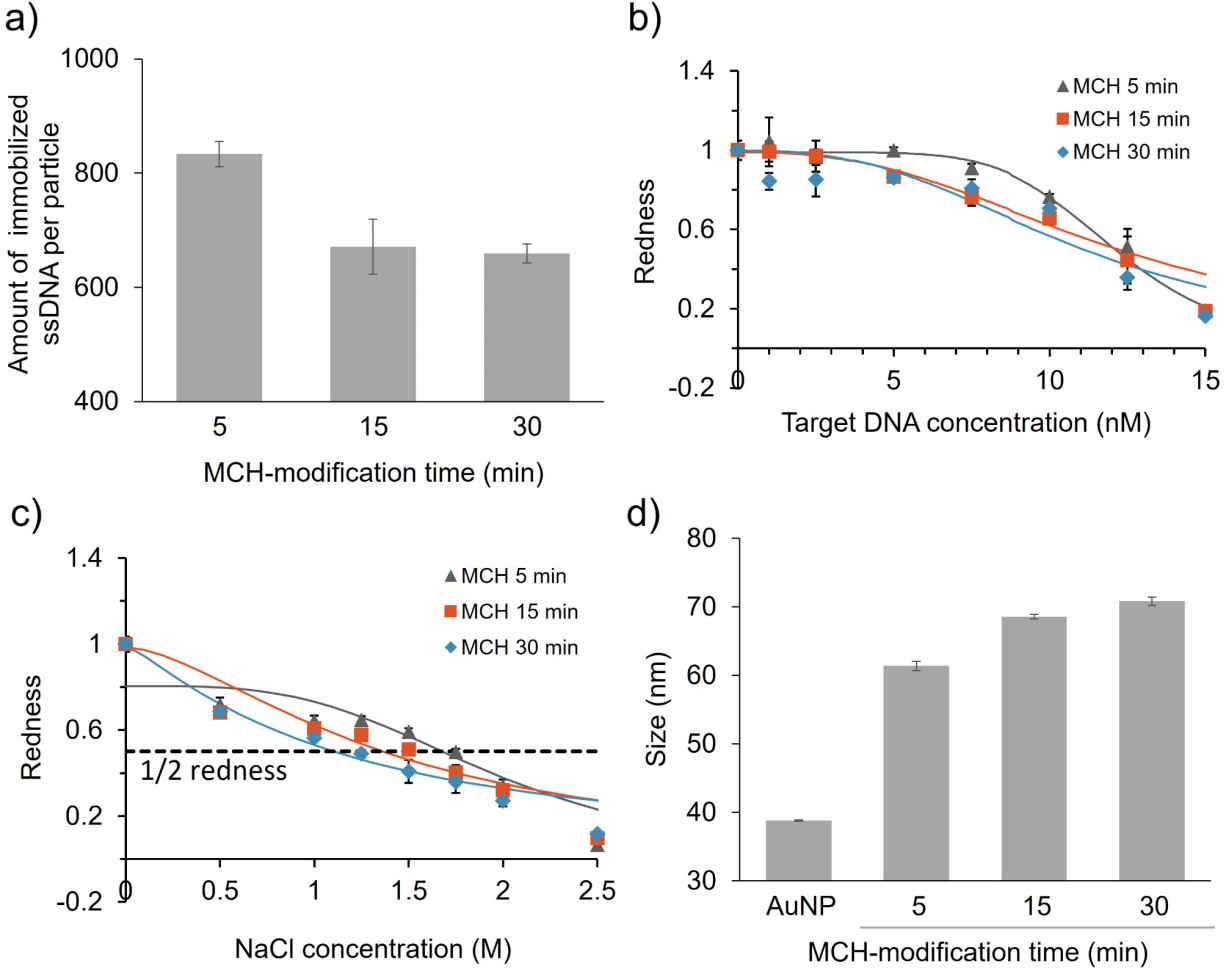
Control of immobilised probe DNA-3 density using MCH and effect on ssDNA-AuNP aggregation. (**a**) Density of probe DNA-3 controlled with MCH. Averaged values from three independent measurements are shown. (**b** and **c**) Effect of immobilised probe DNA density on detection sensitivity (**b**) and dispersion stability of ssDNA-AuNPs against salt (**c**). Normalised redness values obtained from solution colour. The redness value of samples without NaCl or target DNA was normalised to 1.0. Averaged values from three different samples are shown. LOD: 9.04 nM (MCH 5 min), 3.84 nM (MCH 15 min), 1.72 nM (MCH 30 min). C½ redness: 1.68 M (MCH 5 min), 1.37 M (MCH 15 min), 1.10 M (MCH 30 min). (**d**) Effect of density on ssDNA-AuNP particle size. Averaged values from three measurements are shown.

We further investigated the immobilised DNA density-dependent aggregation of ssDNA-AuNPs through another surface modification that eliminates the interaction between the probe DNA backbone and AuNP surface. Alkanethiols have thiol groups that allow binding to the Au surface and displacement of the immobilised DNA. Treatment with 6-mercapto-1-hexanol (MCH), a short alkanethiol with a terminal hydroxy group, inhibits the interaction between the DNA backbone and Au surface through the formation of a MCH monolayer, causing immobilised DNA to stand upright^24,25^. In this study, we simultaneously inhibited the adsorption of the probe DNA backbone to the surface of AuNPs and controlled the density of the immobilised probe DNA using MCH. The immobilised density of all probe DNA decreased in an MCH modification time-dependent manner (Fig. 6a, type-3, ESI; Fig. S5, type-1 and type-2). Interestingly, decreasing DNA density by MCH improved the detection sensitivity of type-3 (LOD: 9.04 nM (MCH 5 min), 3.84 nM (MCH 15 min), 1.72 (MCH 30 min)) (Fig. 6b), unlike type-3 treated by EG (Fig. 5b). And decreasing DNA density by MCH also reduced the dispersion stability against salt (Fig. 6c), unlike type-3 treated by EG (Fig. 5c). Notably, the aggregation behaviour of type-1 and type-2 were similar between the MCH treatments (ESI, Fig. S6) and EG treatments (Figs. 2 and 3). The adsorption of the probe DNA backbone onto the Au surface and subsequent structural changes observed only for low-density type-3 suggested that the structure of the probe DNA affected the dispersion stability and detection sensitivity of the ssDNA-AuNPs. This is consistent with the particle size measurements: the particle size of type-3 ssDNA-AuNPs increased with the MCH modification, suggesting an extension of the immobilised probe DNA (Fig. 6d). A comparison of density control methods also suggested that controlling the DNA density on AuNPs by EG treatment may result in preserved structure of probe DNA without an effect of surface modification.

To further discuss relationship between the effect of the density of immobilized probe DNAs on the detection sensitivity and their structure such as type-2, other probe DNAs that shows steric structures were examined; (1) rigid stem-loop probe DNA (rigid stem probe) in which the stem structure was strengthened by changing to G-C pairs from A-T pairs at two locations (ESI, Fig. S1(b)); (2) G-quadruplex (G4) probe DNA (G4 probe) (Table 3). For G4 probe, the oligo DNA which was shown to take G4 structure on AuNPs was used^37^.

**Table 3:** Sequences of probe and target DNAs

DNA	Sequence
rigid stem probe DNA	5’-SH-(CH_2_)_6_-AT**C GAG GTC G**GA GCT **CGA CCT CG**C-3’
G4 probe DNA	5’-SH-(CH_2_)_6_-AAA AAT CTC GGT TGG TGT GGT TGG-3’
rigid stem target DNA	5’-GCG AGG TCG AGC TCC GAC CTC GAT-3’
G4 target DNA	5’-CCA ACC ACA CCA ACC GAG ATT TTT-3’

The sequences considered to form the stem structure are bold.

The amount of rigid stem probe DNA immobilized on AuNPs decreased with EG concentration and MCH modification in a time-dependent manner (ESI, Fig. S7(a)), consistent with the type-1, -2 and − 3 results. In both cases, the decrease in the DNA density by EG and MCH worsened the detection sensitivity of target ssDNA toward the rigid stem probe (ESI, Fig. S7(b) and (c)), and decreased its dispersion stability against salt (ESI, Fig. S7 (d) and (e)). These results were supported by the LOD values (89 nM (EG 0 mM), 137 nM (EG 2 mM), 2200 nM (EG 6 mM), 1940 nM (MCH 5 min), and 2180 nM (MCH 15 min)) for density controlled with EG and MCH, respectively. Results using G4 probe showed similar trend; the amount of G4 probe immobilized on AuNPs decreased in an EG concentration and MCH modification time-dependent manner (ESI, Fig. S8(a)), and the detection sensitivity worsened with decreasing immobilization density (ESI, Fig. S8(b) and (c)) (LOD = 14.3 nM (EG 0 mM), 105 nM (EG 2 mM), 112 nM (EG 6 mM), 43.6 nM (MCH 5 min), 112 nM (MCH 15 min), 587 nM (MCH 30 min)). These results indicate that even in probe DNAs that adopt tertiary structures such as stem-loop and G-quadruplex structures, different in sequence from type-2, the detection sensitivity was worsened with decreasing DNA density, suggesting that this aggregation tendency can be applicable for other ssDNAs with different sequences or structures.

## Conclusions

This study investigated the immobilised probe DNA density-dependent aggregation behaviour of AuNPs modified with different DNA sequences. We found that a decrease in probe DNA density worsened detection sensitivity for probe DNA retaining a rigid stem structure, unlike that for probe DNA with a linear structure. Interestingly, controlling immobilised DNA density using EG had different effects on ssDNA-AuNP aggregation compared to those using MCH for type-3. Our results suggest that both dispersion stability against salt and duplex formation ratio are important for improved detection sensitivity. We also propose that the steric structure of the probe DNA has a strong effect on AuNP aggregation at lower densities. This study provides an important basis for determining the optimum DNA immobilisation conditions for developing ssDNA-AuNP sensors.

## Methods

### Materials

AuNPs (40 nm) were obtained from BBI solution (Cardiff, UK). Probe ssDNAs (Tables 1, 2 and 3; probe DNA-1, probe DNA-2, probe DNA-3, rigid stem probe DNA and G4 probe DNA) were purchased from Integrated DNA Technologies (Coralville, IA, USA). Complementary ssDNAs (Tables 1, 2 and 3; target DNA-1, target DNA-2, target DNA-3, rigid stem target DNA and G4 target DNA) and non-thiolated ssDNAs were purchased from Eurofins Genomics (Tokyo, Japan). Dithiothreitol (DTT), and EG were purchased from Wako (Osaka, Japan). MCH was purchased from Sigma-Aldrich (St. Louis, MO, USA). NAP-5 columns (Sephadex G-25 DNA grade) were purchased from Cytiva (Little Chalfont, UK). All aqueous solutions were prepared using Milli-Q (MQ) water (Merck Millipore, Burlington, VT, USA). The room temperature of the experimental room was maintained at 25 °C all the time.

### Preparation of ssDNA-AuNPs

Probe ssDNA was immobilised on the AuNP surface via a thiol-Au bond using the freezing method, as previously described^27^. Briefly, thiol-ssDNA was treated with DTT, purified using a NAP-5 column, mixed with AuNPs (DNA: AuNP concentration ratio of 40,000:1), and frozen at − 80 °C for 40 min^10^. EG was added to the mixture at various concentrations (2 and 6 mM) before freezing to control the amount of immobilised probe DNA. After thawing, the unreacted probe ssDNA was removed by centrifugation, and the ssDNA-AuNPs were redispersed in MQ water at 750 pM as a stock solution.

MCH modification of ssDNA-AuNPs was performed as follows: The stock ssDNA-AuNPs solution (750 pM) was incubated with 1 mM MCH at 50 °C for 5, 15, or 30 min. The unreacted MCH was removed by centrifugation and the MCH-ssDNA-AuNPs were redispersed in MQ water at 750 pM as a stock solution. MCH-unmodified samples were heated for 5 min and washed similarly to the MCH-modified samples.

The amount of immobilised ssDNA was estimated as previously described^14^. Briefly, 10 mM DTT was added to the ssDNA-AuNPs solution and incubated at 25 °C for 48 h. After centrifugation at 15,000 rpm for 10 min, the concentration of probe ssDNA in the supernatant was quantified using a QuantiFluor ssDNA System (Promega, Madison, WI, USA). Non-thiolated ssDNAs were used as standard samples.

### Colorimetric evaluation of ssDNA-AuNP dispersion stability against salt

Various concentrations (0–5 M) of NaCl (6 µL) were added to 500 pM ssDNA-AuNP solution (4 µL) and incubated for 60 min at room temperature. Images were captured with a digital camera under constant conditions using a dark box and LED illumination (Fujicolor LED viewer pro, Fujifilm, Tokyo, Japan), and colour analysis was performed using ImageJ software (Ver. 1.52i, https://imagej.net/ij/)^38^. Briefly, images were split into red, green, and blue components. The redness of the solution was quantified as (red – (green + blue) / 2), based on the intensity of each colour component, as previously described^10^. The redness value of samples without NaCl was normalised to 1.0. The averaged values from the pictures of the three different sample tubes that were taken at the same time were used. It was also confirmed in advance that the redness values could reflect the change in the color of AuNP solution upon aggregation as the OD_630_/OD_530_ ratio obtained from UV- VIS spectra^10^. For the quantitative evaluation of the stability against salt, data was fitted with an equation that is used to obtain LOD values as show below, and C_½ redness_ values was calculated as the concentration that gives lower value than ½ redness line (= 0.5).

### Colorimetric detection of target ssDNA using ssDNA-AuNPs

Various concentrations of target ssDNA solubilized in MQ water (4 µL) were added to 500 pM ssDNA-AuNPs solution in MQ water (4 µL) and incubated for 10 min at room temperature. We added 5 M NaCl (2 µL, for probe-2 and rigid-stem probe: 2.5 M) to the sample before incubation for 60 min at room temperature for type-1 and-3, and at 4 °C for type-2 and rigid-stem (for enhanced hybridization), respectively. For G4 probe, target DNA was added to G4-AuNPs in the presence of 100 mM NaCl to ensure formation of G4 structure^39,40^, and incubated for 60 min at 4 °C. Images were captured using the digital camera, and the RGB values of the colour images were determined as described for dispersion stability. LOD was evaluated using 3σ criterion method, where σ denotes the standard deviation of zero-concentration background data as previously reported^29^. The data was fitted by with a four-parameter logistic function: y = d+(a-d)/[1+(x/c)^b^], where a, b, c and d are fitting parameters. These parameters were optimized by nonlinear least-square regression weighted by the reciprocal of the square of standard deviation of each datum point. The LOD was calculated from the fitting equation as the concentration that gives lower value than 3σ line (= background + 3σ).

### Particle size and zeta potential of ssDNA-AuNPs

The size and zeta potential of 75 pM AuNP samples diluted in MQ water were evaluated using a Zetasizer-Nano ZS (Malvern Worcestershire, UK).

### Evaluation of duplex formation ratio

ssDNA-AuNP solution (750 pM) was centrifuged at 5,000 rpm and 4 °C for 30 min, and the supernatant was replaced with 450 µL PBS buffer including 0.01% Tween-20. The washing process was repeated and the ssDNA-AuNPs were redispersed in PBS at 375 pM. After the samples were diluted to 200 pM with PBS, FAM-ssDNA was added to the ssDNA-AuNPs (ssDNA-AuNP: FAM-ssDNA concentration ratio, 1: amount of immobilised DNA on each AuNP) and incubated for 1 h at 4 °C for hybridisation. The washing process was repeated twice to remove non-hybridised FAM-ssDNA, and the dsDNA-AuNPs were redispersed in PBS at 200 pM. An excess amount of complementary ssDNA was added to the samples (dsDNA-AuNP: complementary ssDNA concentration ratio, 1:2500) before incubation at 90 °C for 1 min to dissociate FAM-ssDNA. The sample was kept at 4 °C for 60 min and centrifuged for 30 min at 15,000 rpm and 4 °C. The concentration of FAM-ssDNA in the supernatant was quantified by measuring the fluorescence (excitation, 494 nm; emission, 520 nm; FP-8600, JASCO, Tokyo, Japan) using FAM-ssDNA as the standard. The amount of hybridised FAM-ssDNA per ssDNA-AuNP was calculated using the FAM-ssDNA and AuNP concentrations. The duplex formation ratio was calculated using the amounts of hybridised ssDNA and immobilised probe ssDNA.

### Statistical analysis

Data were statistically analysed using Student’s t-test.

## Electronic supplementary material

Below is the link to the electronic supplementary material.

Supplementary Material 1

## Data Availability

Data is provided within the manuscript or supplementary information files.

## Abbreviations

AuNPs: Gold nanoparticles
ssDNA: Single-stranded DNA
Probe DNA: Immobilised single-stranded DNA
Target DNA: Target complementary single-stranded DNA
ssDNA-AuNPs: Single-stranded DNA-immobilised gold nanoparticles
EG: Ethylene glycol
Freeze method: Freeze-thaw single-stranded DNA-immobilised gold nanoparticles synthesis method
Probe DNA-1: Complementary sequence to target DNA-1
Target DNA-1: micro RNA 145
miR: micro RNA
Probe DNA-2: Complementary sequence to target DNA-2
Target DNA-2: Target DNA of probe DNA-2
Type-1: Probe DNA-1-immobilised gold nanoparticles
Type-2: Probe DNA-2-immobilised gold nanoparticles
Probe DNA-3: Complementary sequence to target DNA-3
LOD: Limit of detection
Type-3: Probe DNA-3-immobilised gold nanoparticles
Target DNA-3: miR125a
MCH: 6-mercapto-1-hexanol
G4: G-quadruplex
Rigid stem probe DNA: Complementary sequence to rigid stem target DNA
Rigid stem target DNA: Target DNA of rigid stem probe DNA
Rigid stem-AuNPs: Rigid stem probe DNA-immobilised gold nanoparticles
G4 probe DNA: Complementary sequence to G4 target DNA
G4 target DNA: Target DNA of G4 probe DNA
G4-AuNPs: G4 probe DNA-immobilised gold nanoparticles
DTT: Dithiothreitol
MQ: Milli-Q
C_½ redness_: NaCl concentration that shows half redness (= 0.5)
dsDNA-AuNPs: Double-stranded DNA-immobilised gold nanoparticles
