# Supplementary material for: Density and structure of DNA immobilised on gold nanoparticles affect sensitivity in nucleic acid detection
Source: Sci Rep. 2025 Mar 10;15:8222. doi: 10.1038/s41598-025-92474-y (PMC11893797; doi:10.1038/s41598-025-92474-y)
Supplement: Supplementary file 1 — Supplementary Material 1 [file 41598_2025_92474_MOESM1_ESM.pdf]

Supplementary Information

Density and structure of DNA immobilised on gold nanoparticles affect sensitivity in nucleic acid detection

Nanami Fukuzumi[1], Gen Hirao[1], Atsushi Ogawa[2], Tsuyoshi Asahi[1], Mizuo Maeda[3], Tamotsu Zako[1*]

[1]Department of Chemistry and Biology, Graduate School of Science and Engineering, Ehime University, 2-5 Bunkyo, Matsuyama, Ehime 790-8577, Japan

[2]Proteo-Science Center, Ehime University, 2-5 Bunkyo, Matsuyama, Ehime 790-8577, Japan

[3]RIKEN Cluster for Pioneering Research, 2-1 Hirosawa, Wako, Saitama 351-0198, Japan

[*]Corresponding author: Tamotsu Zako (zako.tamotsu.us@ehime-u.ac.jp)

Table of Contents

Figure S1 Predicted structures of stem-loop probe DNAs.

Figure S2 Significance of the difference between samples with different probe DNA densities.

Figure S3 Evaluation of the detection sensitivity using the 3σ criterion method.

Figure S4 Zeta potential of ssDNA-AuNPs following control of immobilised DNA density.

Figure S5 Effect of MCH on density of immobilised probe DNA.

Figure S6 Effect of controlling immobilised ssDNA with MCH on ssDNA-AuNP aggregation behaviour.

Figure S7 Effect of controlling immobilised ssDNA with EG or MCH on rigid stem-AuNP aggregation behaviour.

Figure S8 Effect of controlling immobilised ssDNA with EG or MCH on G4-AuNP aggregation behaviour.

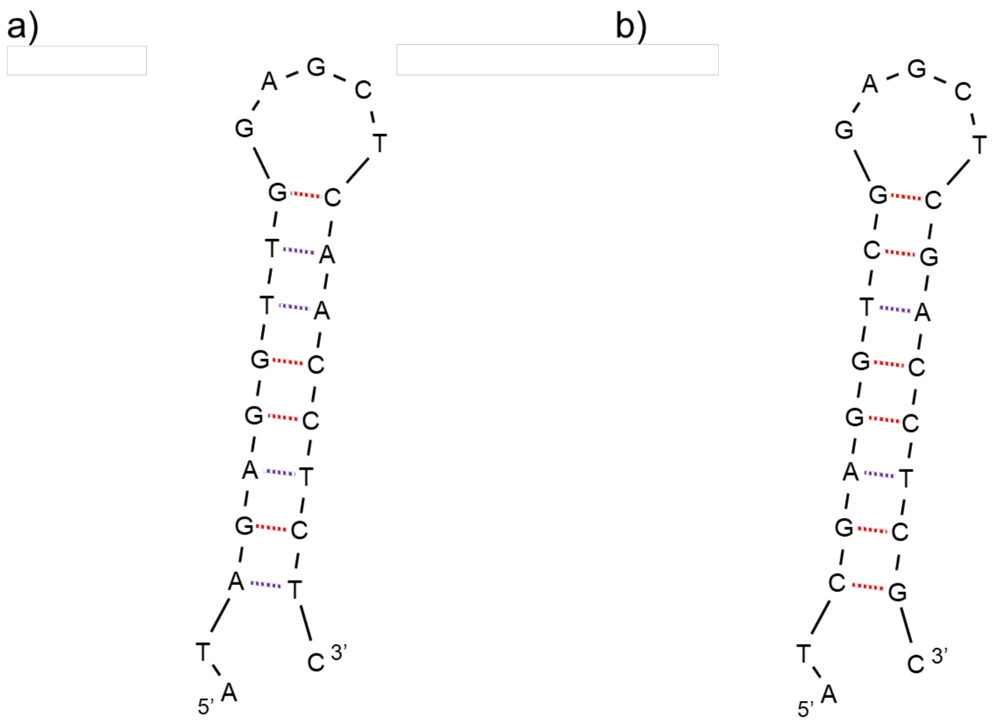

Figure S1 Predicted structures of stem-loop probe DNAs. Structures of probe DNA-2 (left) and rigid stem probe DNA (right) were predicted using OligoAnalyzer™ Tool (Integrated DNA Technologies, https://sg.idtdna.com/page). The most stable structure was shown.

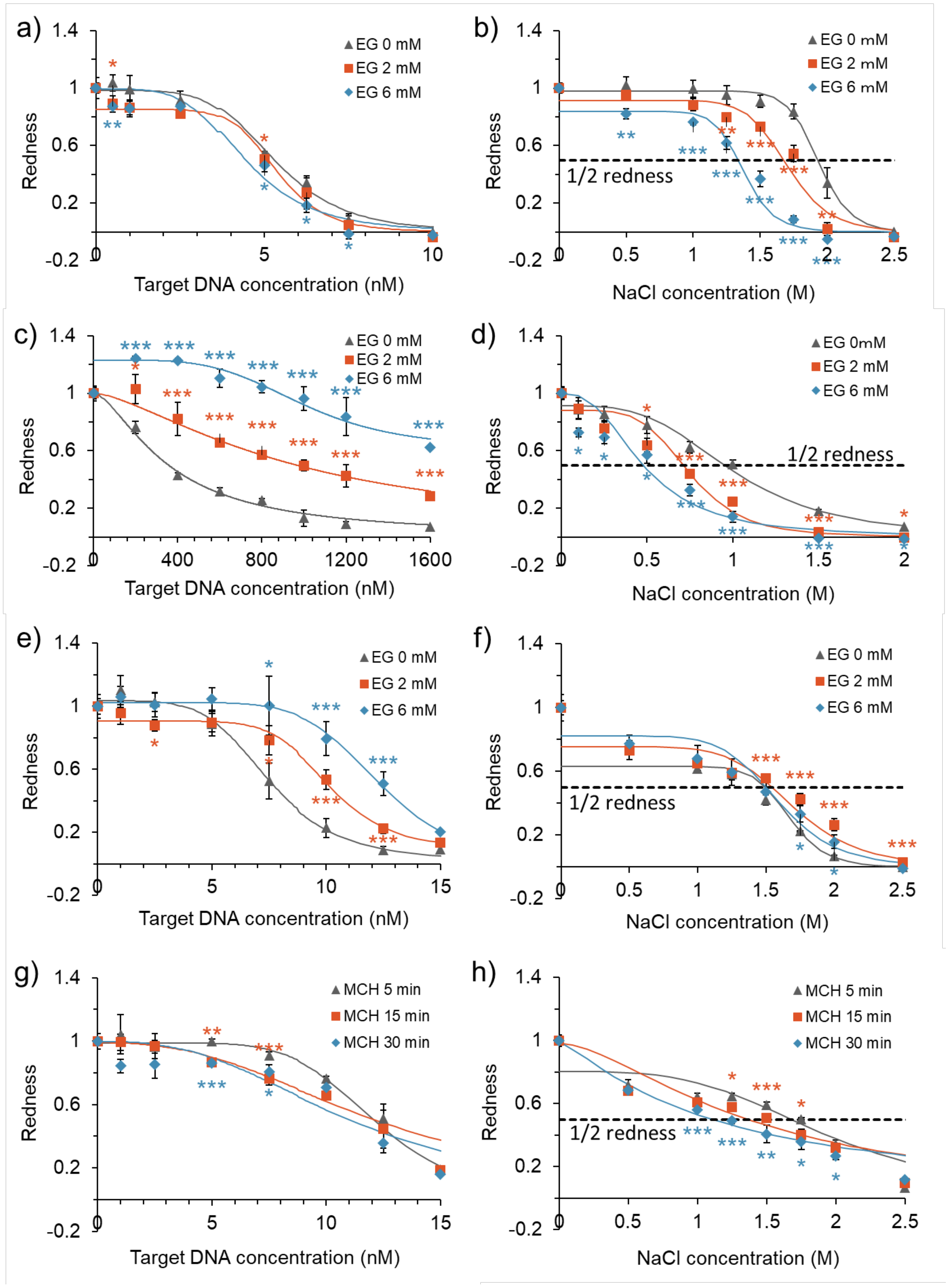

Figure S2 Significance of the difference between samples with different probe DNA densities. The student's t-test is used to determine the significance of the difference in the detection sensitivity (left) and the dispersion stability against salt (right) between the samples with different probe DNA densities. (*, $p < 0.05$, **, $p < 0.01$, ***, $p < 0.005$). The calculated p-values were shown in each graph as orange (EG 0 mM-2 mM, MCH 5 min-15 min) and blue (EG 0 mM-6 mM, MCH 5 min-30 min) star [detection sensitivity, type-1 EG(a), type-2 EG(c), type-3 EG(e), type-3 MCH(g); dispersion stability, type-1 EG (b), type-2 EG (d), type-3 EG (f), type-3 MCH (h)].

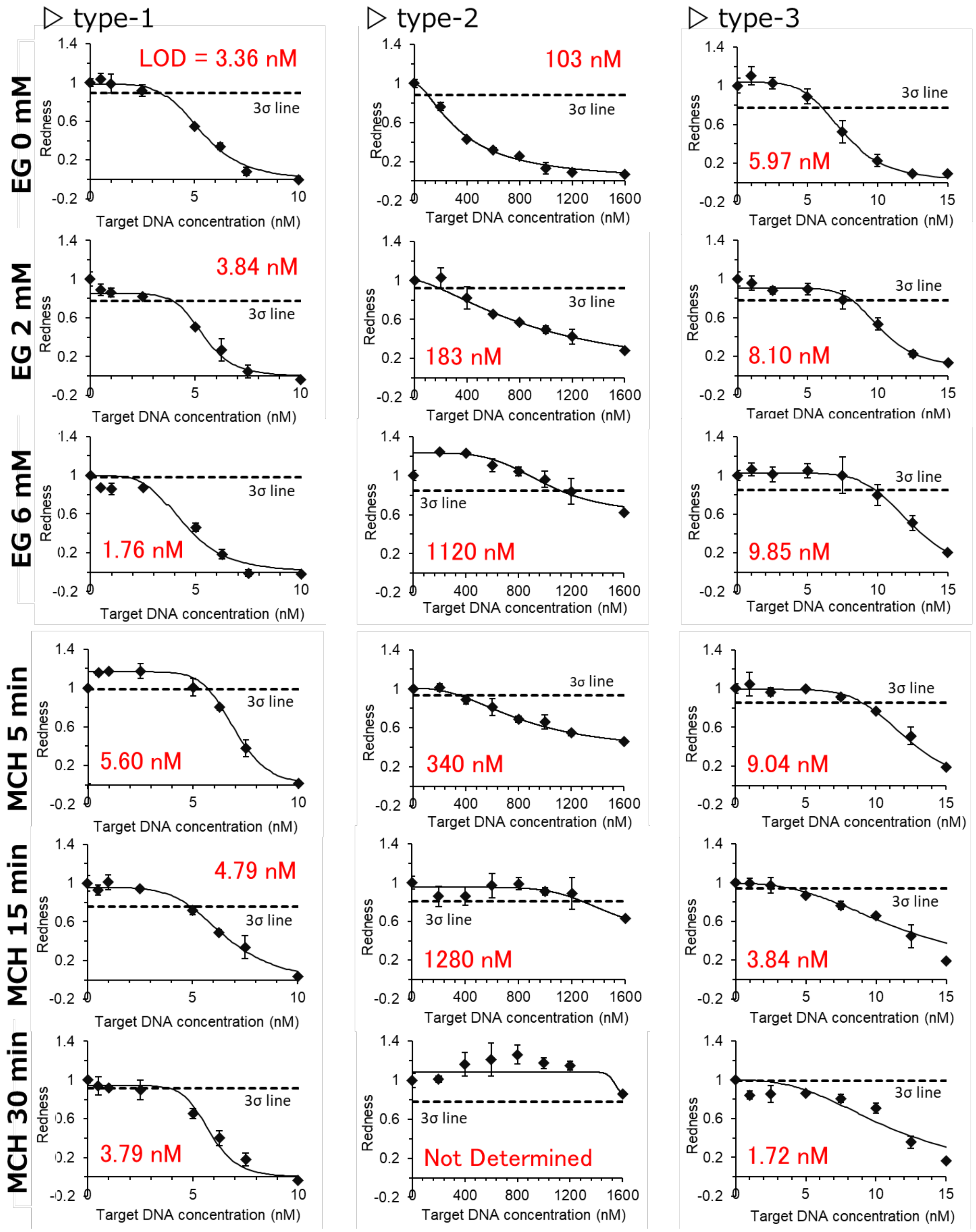

Figure S3 Evaluation of the detection sensitivity using the 3σ criterion method. Normalised redness values for type-1 (left), type-2 (centre) and type-3 (right) AuNP samples with fitting curves and 3σ lines were shown. The amounts of immobilized probe DNAs were regulated using EG (upper) or MCH (lower). The limit of detection (LOD) values evaluated using the 3σ criterion method were also shown.

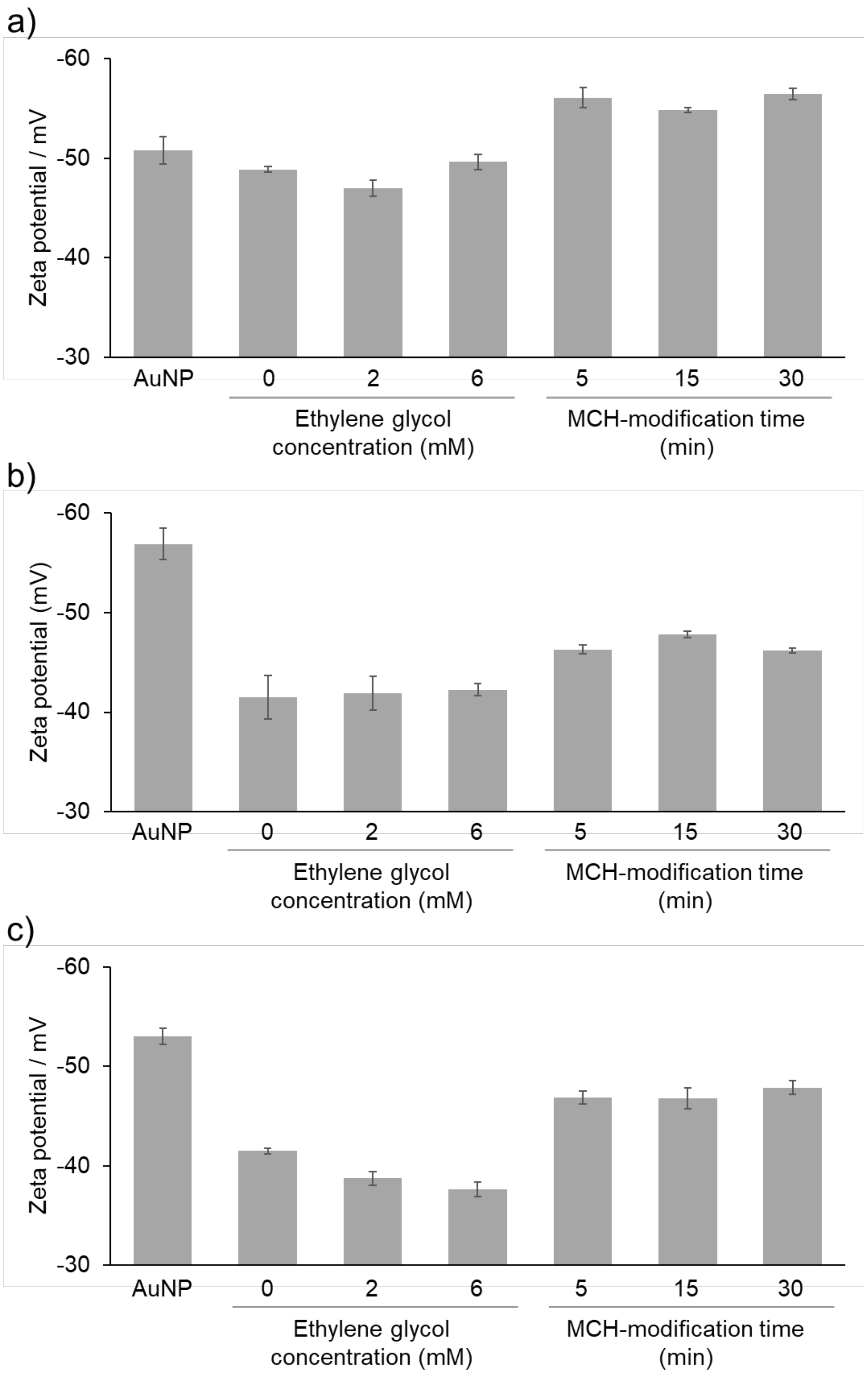

Figure S4 Zeta potential of ssDNA-AuNPs following control of immobilised DNA density. Zeta potential of 75 pM unmodified AuNPs and ssDNA-AuNPs [type-1 (a), type-2 (b), and type-3 (c)] with amount of immobilised DNA controlled using EG and MCH diluted in MQ water was measured using Zetasizer-Nano ZS. Averaged values of three measurements are shown.

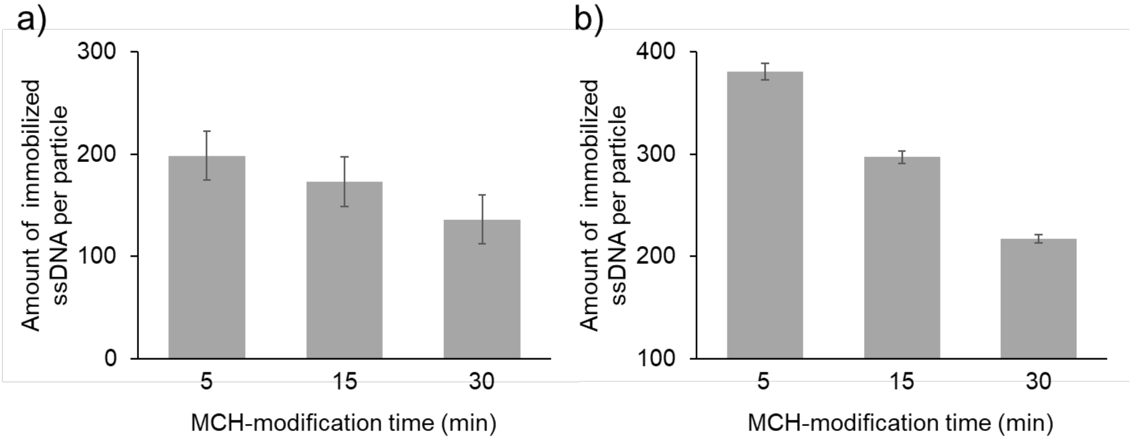

Figure S5 Effect of MCH on immobilised probe DNA density. The amounts of immobilised probe DNA-1 (a) and probe DNA-2 (b) with increasing MCH modification time were averaged from three independent measurements.

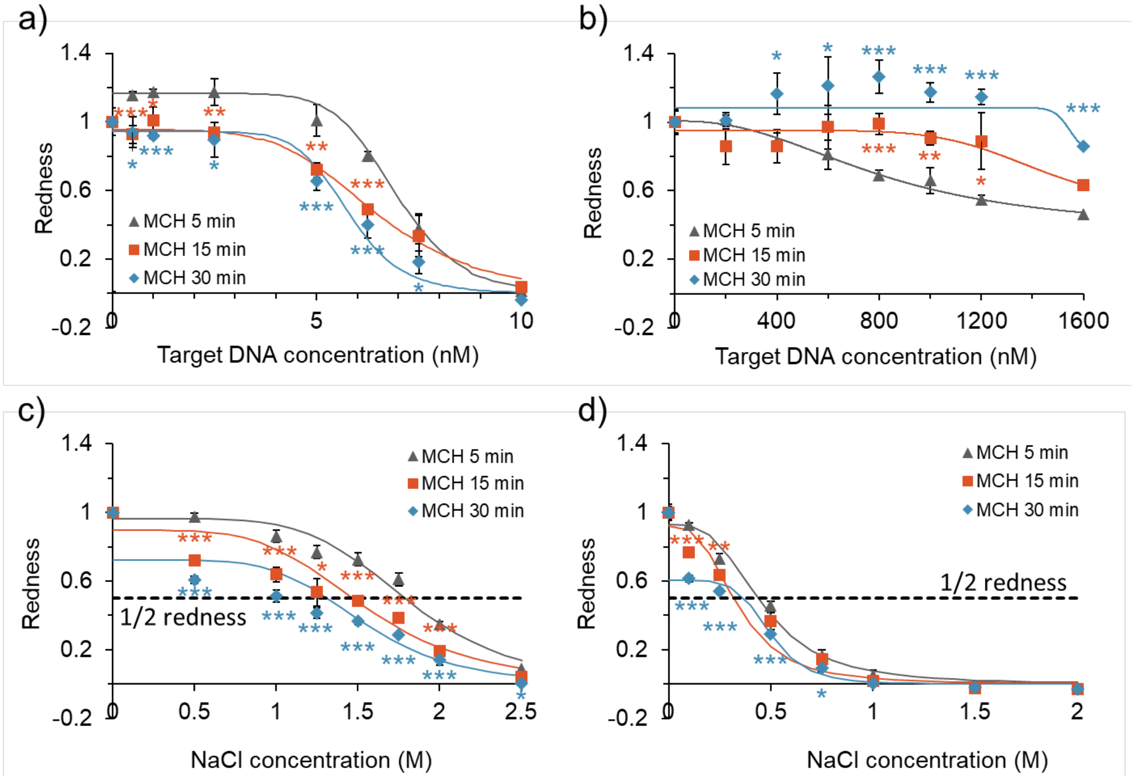

Figure S6 Effect of controlling immobilised ssDNA with MCH on type-1/type-2 ssDNA-AuNP aggregation behaviour. (a and b) Effect of immobilised probe DNA density on the detection sensitivity of ssDNA-AuNPs for target ssDNA. (c and d) Effect of immobilised probe DNA density on dispersion stability against salt. Normalised redness values for type-1 (a and c) and type-2 (b and d) samples. The redness value of samples without target ssDNA or NaCl was normalised to 1.0. Averaged values of three different samples are shown. The calculated significant differences are shown in each graph as orange (EG 0 mM-2 mM, MCH 5 min-15 min) and blue (EG 0 mM-6 mM, MCH 5 min-30 min) star (*, $p < 0.05$, **, $p < 0.01$, ***, $p < 0.005$). For type-1, a lower density was associated with higher detection sensitivity (LOD, 5.60 nM (MCH 5 min), 4.79 nM (MCH 15 min), 3.79 (MCH 30 min)) and lower dispersion stability ($C_{½\ redness}$, 1.79 M (MCH 5 min), 1.47 M (MCH 15 min), 1.31 M (MCH 30 min)) (a and c). On the other hand, for type-2, a lower density was associated with lower detection sensitivity (LOD, 340 nM (MCH 5 min), 1280 nM (MCH 15 min), not determined (MCH 30 min)) and dispersion stability ($C_{½\ redness}$, 0.438 M (MCH 5 min), 0.318 M (MCH 15 min), 0.369 M (MCH 30 min)) (b and d).

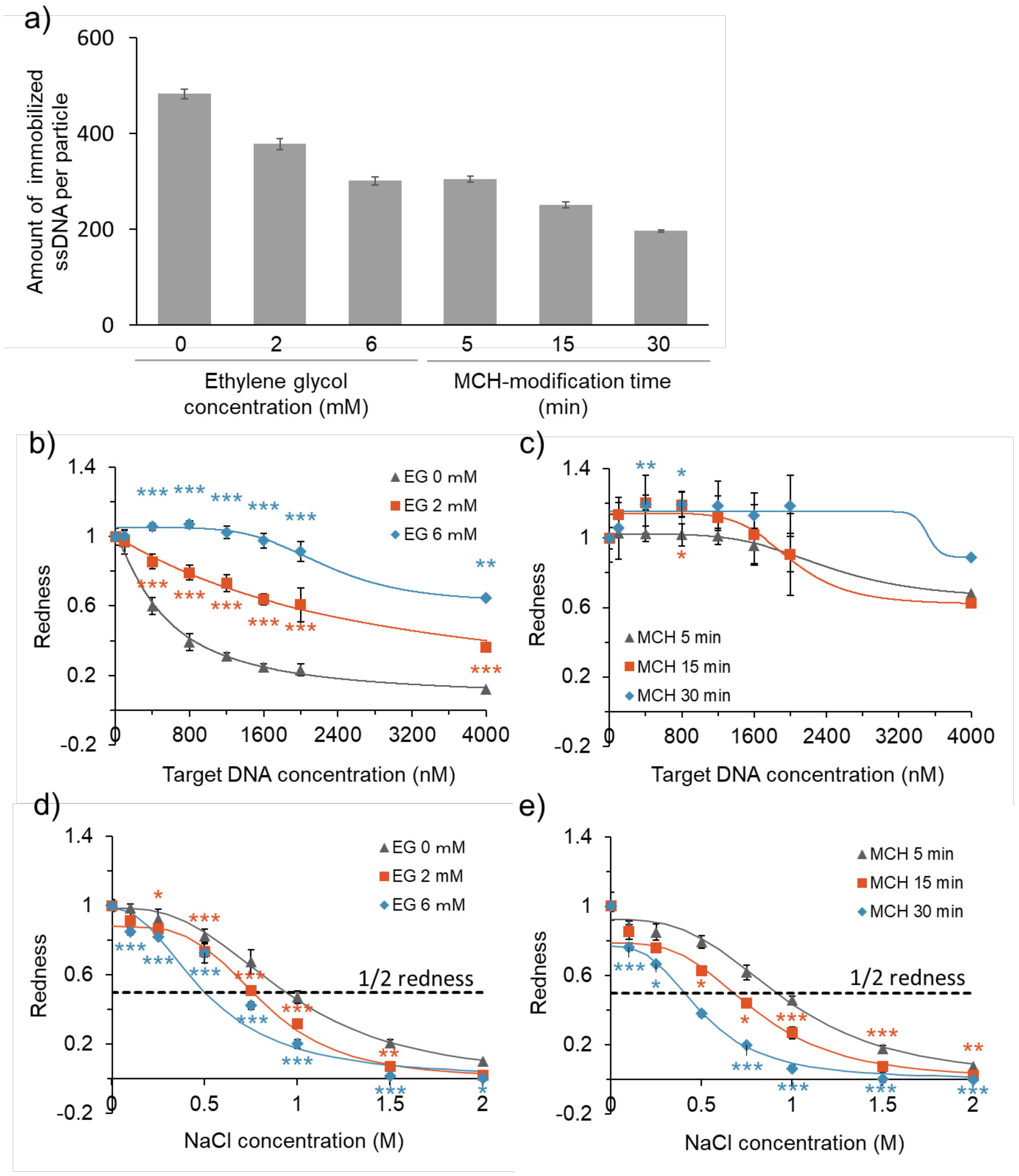

Figure S7 Effect of controlling immobilised ssDNA on rigid stem-AuNP aggregation behaviour. (a) Density of rigid stem probe DNA controlled with EG and MCH. Averaged values from three independent measurements are shown. (b and c) Effect of immobilised probe DNA density on the detection sensitivity of rigid stem-AuNPs for target ssDNA [controlled with EG(b), MCH(c)]. Various concentrations of target ssDNA solubilized in MQ water (4 µL) were added to 500 pM rigid stem-AuNPs solution in MQ water (4 µL) and incubated for 10 min at room temperature. We added 2.5 M NaCl (2 µL) to the sample before incubation for 60 min at 4°C for enhanced hybridization. (d and e) Effect of immobilised probe DNA density on dispersion stability against salt [controlled with EG(d), MCH(e)]. The calculated significant differences are shown in each graph (b)-(e) as orange (EG 0 mM-2 mM, MCH 5 min-15 min) and blue (EG 0 mM-6 mM, MCH 5 min-30 min) star (*, $p < 0.05$, **, $p < 0.01$, ***, $p < 0.005$). The amount of rigid stem probe DNA immobilised on AuNPs decreased in an EG concentration- and MCH modification time-dependent manner (a). A lower density was associated with higher detection sensitivity (LOD, 89.0 nM (0 mM), 137 nM (2 mM), 2200 nM (6 mM), 1940 nM (MCH 5 min), 2180 nM (MCH 15 min), not determined (MCH 30 min)) (b and c) and lower dispersion stability (d and e) ($C_{½\ redness}$, 0.936 M (0 mM), 0.757 M (2 mM), 0.500 M (6 mM), 0.913 M (MCH 5 min), 0.677 M (MCH 15 min), 0.404 M (MCH 30 min)).

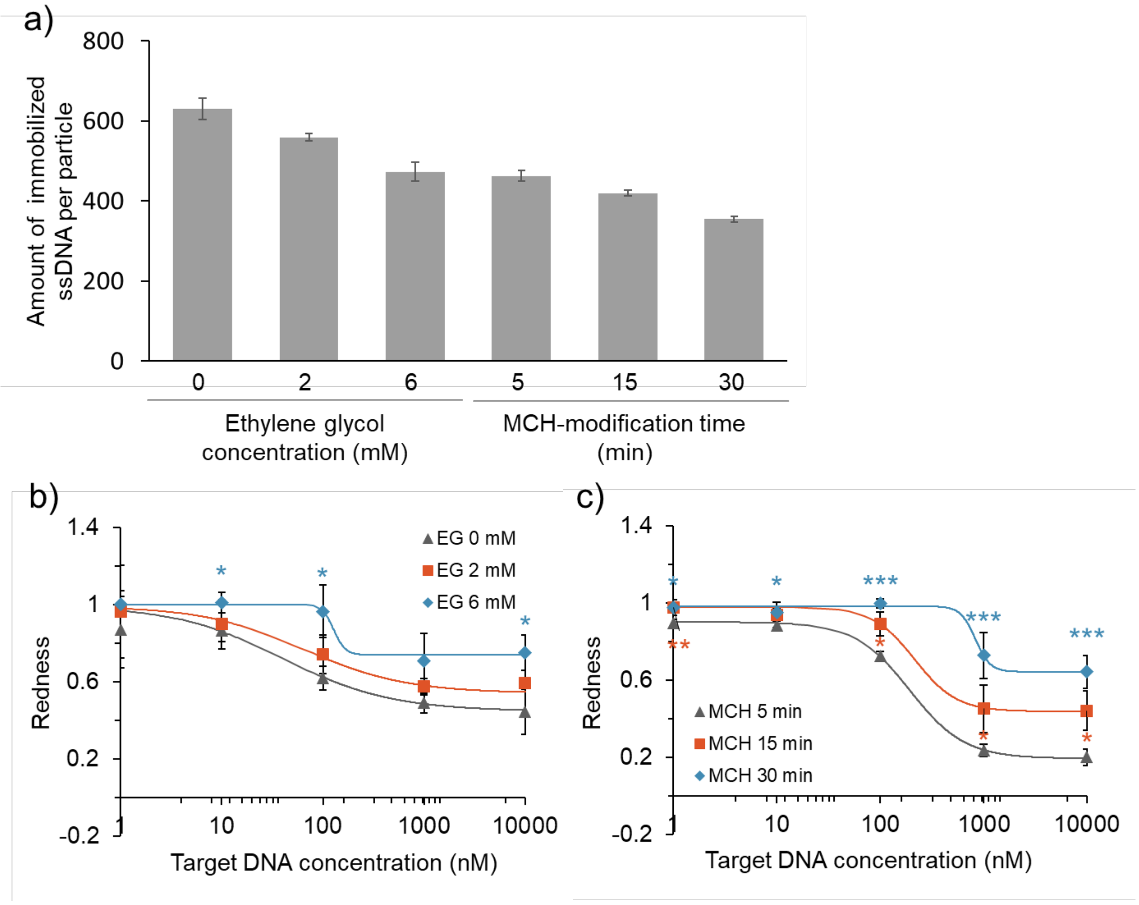

Figure S8 Effect of controlling immobilised ssDNA on G4-AuNP aggregation behaviour. (a) Density of G4 probe DNA controlled with EG and MCH. Averaged values from three independent measurements are shown. (b and c) Effect of immobilised probe DNA density on the detection sensitivity of G4-AuNPs for target ssDNA [controlled with EG(b), MCH(c)]. 0.5 M NaCl (2 µL) were added to 500 pM G4-AuNPs solution in MQ water (4 µL) and incubated for 5 min at room temperature for enhanced G4 structure formation. We added various concentrations of target ssDNA solubilized in MQ water (4 µL) to the sample before incubation for 60 min at 4°C for enhanced hybridization. The calculated significant differences are shown in each graph as orange (EG 0 mM-2 mM, MCH 5 min-15 min) and blue (EG 0 mM-6 mM, MCH 5 min-30 min) star (*, $p < 0.05$, **, $p < 0.01$, ***, $p < 0.005$). The amount of G4 probe DNA immobilised on AuNPs decreased in an EG concentration- and MCH modification time-dependent manner (a). A lower density was associated with higher detection sensitivity (LOD, 14.3 nM (0 mM), 105 nM (2 mM), 112 nM (6 mM), 43.6 nM (MCH 5 min), 112 nM (MCH 15 min), 587 n (MCH 30 min)) (b and c).
